# Presentation and Management of Progestogen Hypersensitivity in a Male Patient in the Emergency Department and Intensive Care Unit

**DOI:** 10.7759/cureus.115235

**Published:** 2026-08-26

**Authors:** Arman Manjikian, Kush Kapadia, Yi McWhorter, Kord Strebel

**Affiliations:** 1 Internal Medicine, HCA Healthcare, MountainView Hospital, Las Vegas, USA; 2 Obstetrics and Gynecology, HCA Healthcare, MountainView Hospital, Las Vegas, USA

**Keywords:** autoimmune progesterone dermatitis, critical care medicine, hormone induced anaphylaxis, male case report, mast cell degranulation, progestogen hypersensitivity

## Abstract

Progestogen hypersensitivity (PH), historically termed autoimmune progesterone dermatitis, is an exceedingly rare condition documented almost exclusively in reproductive-age women. Although the pathophysiology remains incompletely understood, affected patients typically develop cutaneous and mucocutaneous findings, bronchospasm, and/or anaphylaxis following exposure to endogenous progesterone or exogenous progestins. Diagnosis rests on clinical history and characteristic physical findings, supported where available by progesterone skin testing, which is not standardized and has limited sensitivity and specificity. Cases in men are exceptionally rare, and the resulting scarcity of literature contributes to frequent misdiagnosis. We report a 56-year-old man with coronary artery disease, prior resection of an intestinal carcinoid tumor, and recurrent documented episodes of PH since 2006. He presented to the emergency department with a diffuse rash, facial angioedema, and nausea, and received intravenous fluids, epinephrine, antihistamines, and corticosteroids. Because his symptoms persisted and further epinephrine dosing was limited by chest pain in the setting of coronary artery disease, he was admitted to the intensive care unit for airway monitoring. Inpatient management consisted of dexamethasone, cromolyn sodium, testosterone cypionate, analgesia, and supportive care, with rapid improvement and discharge on hospital day three. This report describes a symptom-directed approach to the management of PH in a male patient in the critical care setting and considers the comorbidities and possible mechanisms that may underlie his presentation. Given the rarity of the condition and the limited literature in men, effective management currently depends on an individualized, symptom-directed strategy.

## Introduction

Progestogen hypersensitivity (PH) is an exceedingly rare disease. Although its true incidence is unknown, a PubMed search for case reports published between 1967 and 2025 using the terms *PH* and *autoimmune progesterone dermatitis* (APD) yielded 84 and 103 case reports, respectively. Only one of these involved a male patient.

In 1947, Zondek and Bromberg were the first to document allergic sensitivity to endogenous hormones in women, naming the condition endocrine allergy [1]. In 1964, Shelley et al. coined the more specific term APD based on cutaneous and mucocutaneous findings associated with the luteal phase of the menstrual cycle, when progesterone levels peak [2]. APD is now considered an outdated term for two reasons: there is no definitive evidence that the reaction is autoimmune, and the term does not encompass non-dermatologic manifestations such as bronchospasm and anaphylaxis. Investigators at Brigham and Women's Hospital therefore proposed the term PH, as progestogen encompasses all hormones that bind the progesterone receptor, including both endogenous progesterone and synthetic progestins [3]. We use both terms interchangeably in this report.

The pathophysiologic mechanism of PH remains unclear. PH primarily affects women of reproductive age, and cases typically demonstrate a close association with the luteal phase of the menstrual cycle, presenting cyclically with cutaneous and mucocutaneous symptoms. PH has also been described in women taking progestin-containing contraceptives and in those receiving progesterone supplementation during in vitro fertilization. Reports in postmenopausal women are exceptional and generally involve exogenous progestogen exposure, and only one male case has been documented, following exposure to a progestin analog [3,4].

Diagnosis of PH rests principally on clinical history and characteristic physical findings. Progesterone skin testing may support the diagnosis but cannot establish it alone: the technique is not standardized, sensitivity and specificity are limited, and irritant or vehicle-related false-positive results are well described [3,5]. Patients often report a cyclical symptom pattern linked to the menstrual cycle, use of exogenous progestogens, a history of steroid use, atopy, or assisted fertilization [5]. Common physical manifestations include dermatitis, urticaria, angioedema, bullous lesions, erythema multiforme, bronchospasm, and anaphylaxis [5,6].

No established treatment guidelines for PH exist. Recommendations from Brigham and Women's Hospital, developed for female patients, are based on goal-directed therapy. First-line options for symptom control include high-dose antihistamines, oral and topical corticosteroids, oral contraceptive pills, omalizumab, and depot leuprolide; desensitization schedules have been proposed for women pursuing in vitro fertilization [3]. No formal recommendations exist for men beyond symptom-directed therapy such as corticosteroids, mast cell stabilizers, anti-immunoglobulin E (IgE) therapy, and epinephrine.

Here, we report a male patient who presented to the emergency department and was treated in an inpatient critical care setting. We emphasize the treatment modalities that relieved his acute symptoms as well as the long-term therapies continued on an outpatient basis. This report does not aim to establish treatment guidelines but to serve as a reference for similar presentations in the critical care setting. It was prepared in accordance with the CARE (CAse REport) guidelines.

## Case presentation

The patient was a 56-year-old Caucasian man with stable coronary artery disease managed medically, with no history of percutaneous coronary intervention or coronary artery bypass grafting, an intestinal carcinoid tumor resected in 2008, and PH with recurrent hypersensitivity episodes since 2006. He presented to the emergency department with a diffuse rash; swelling of the lips, tongue, and eyelids; and nausea. He reported that he could feel the allergy starting the day prior but was unable to provide further detail.

He had previously been evaluated by immunology specialists, who diagnosed autoimmune progesterone dermatitis based on a positive progesterone skin-prick test that itself provoked a systemic reaction requiring emergency transfer to the emergency department. That testing was performed at an outside institution, and the records were not available to the present authors; the progesterone concentration, the vehicle used, whether positive and negative controls were included, and the timing and magnitude of the wheal-and-flare response could not therefore be verified. He was subsequently placed on chronic corticosteroids, which were discontinued after he developed steroid-induced retinopathy and steroid-induced insulin-resistant diabetes mellitus. His primary care provider then started testosterone cypionate 200 mg intramuscularly every 10 days and cromolyn sodium 200 mg orally four times daily. He was not taking any hormone blockers. He reported that his most recent testosterone injection, approximately six days before presentation, was incomplete because the syringe plunger malfunctioned.

In the emergency department, he received a 1 L normal saline bolus, hydroxyzine 50 mg orally, epinephrine 0.3 mg intramuscularly (1:1,000), and dexamethasone 10 mg intravenously. After three hours of observation per department protocol, he demonstrated worsening facial swelling and erythema without airway compromise, and an additional 0.5 mg of intramuscular epinephrine was administered. Shortly thereafter, he reported chest pain, after which no further epinephrine was given; he received dexamethasone 6 mg intramuscularly and hydroxyzine 25 mg orally. Whether any additional agent was administered at that time could not be verified from the records available to the authors. A repeat electrocardiogram, obtained because of his history of coronary artery disease, was unremarkable. At no point during his emergency department or intensive care unit (ICU) course were hypotension, hypoxemia, wheeze, or stridor documented. He was admitted to the ICU for close airway and cardiac monitoring given the persistence and severity of his mucocutaneous symptoms and his need for further epinephrine in the setting of coronary artery disease.

**Figure 1:**
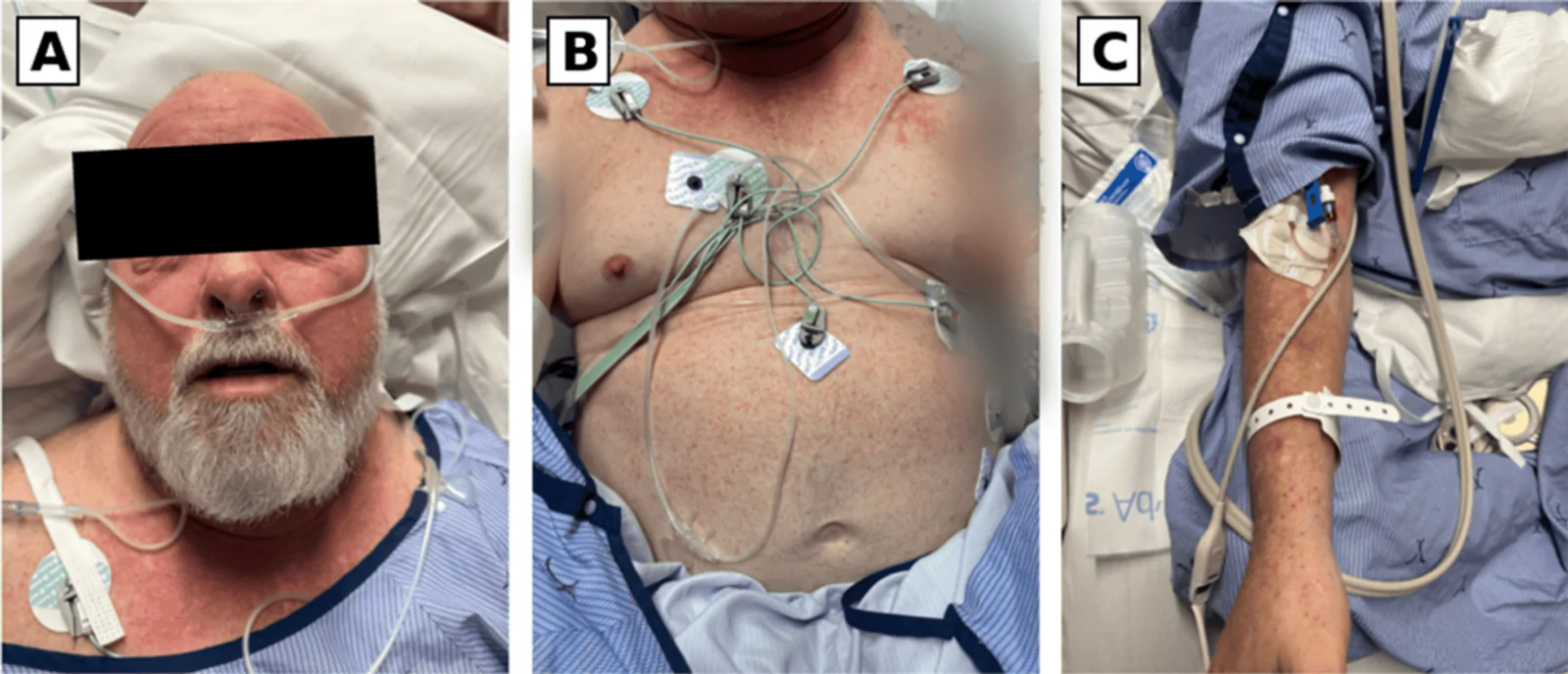
Erythema and papular rash involving the face, scalp, neck, abdomen, and bilateral upper extremities. (A) Facial erythema with labial angioedema. (B) Confluent erythema with pinpoint papular rash across the chest and abdomen. (C) Papular rash involving the upper extremity. To protect patient identity, a black bar has been applied across the eyes and eyebrows in panel A, and identifying tattoos have been blurred in panel B; the bar obscures the periorbital angioedema described in the text. No other alterations to the images were made.

On ICU admission, he had persistent erythema involving the scalp, face, neck, chest, back, abdomen, and bilateral upper extremities, with a pinpoint papular rash most prominent on the back and abdomen. The bilateral lower extremities were unaffected. He also reported swelling of the eyelids, tongue, and lips but denied dyspnea or difficulty managing secretions (Figures 1A-1C). He described the rash as a continuous slapped-sunburn sensation, and ice packs applied to the affected areas provided substantial subjective relief. He was started on dexamethasone and hydromorphone for inflammation and pain control and was restarted on his home regimen of cromolyn sodium 200 mg orally four times daily and testosterone cypionate 200 mg intramuscularly. By hospital day 3, he was cleared for discharge on an oral dexamethasone taper, with near-complete resolution of his symptoms.

With respect to formal diagnostic criteria, the episode was characterized by acute onset of skin and mucosal involvement accompanied by nausea. Neither respiratory compromise nor reduced blood pressure was documented, and his gastrointestinal symptoms were limited to nausea rather than the severe symptoms that would satisfy the gastrointestinal limb of current criteria [7]. The documented findings therefore do not fulfill accepted diagnostic criteria for anaphylaxis, and we describe this episode as a severe mucocutaneous hypersensitivity reaction throughout this report, notwithstanding his documented history of anaphylactic-type reactions and the systemic reaction provoked by his earlier skin-prick testing.

## Discussion

Pathophysiology of PH

Although the exact pathophysiologic mechanism of PH is unclear, the reaction likely results from mast cell degranulation. Two main pathways for mast cell degranulation exist: IgE-mediated (allergic) and non-IgE-mediated (pseudoallergic). It remains uncertain which pathway predominates, or whether both occur simultaneously, but each ultimately results in the release of the mediators responsible for the physical symptoms and manifestations of PH [5,6,8]. Notably, several cases have demonstrated circulating progesterone-specific IgG antibodies with immune-complex formation, consistent with a type III hypersensitivity reaction, or delayed skin-test reactions consistent with a type IV reaction [9,10]. Others have proposed that patients become sensitized to progestogens through cross-sensitization with corticosteroids and other sex hormones [11]. The exact pathophysiology of PH therefore remains under debate.

In IgE-mediated mast cell degranulation, antigen-presenting cells such as dendritic cells capture and present allergen to type 2 helper T cells upon primary exposure [8]. These cells release the cytokines interleukin (IL)-4, IL-5, and IL-13, stimulating memory B cells to produce allergen-specific IgE antibodies. Upon subsequent allergen exposure, IgE binds the allergen and cross-links FcεRI receptors on mast cells, triggering degranulation. This produces symptoms such as urticaria, angioedema, vomiting, diarrhea, and hypotension [8]. When mucocutaneous symptoms coexist with bronchoconstriction or hypotension, the reaction meets the criteria for anaphylaxis [7]. Common triggers include foods such as peanuts, eggs, and shellfish, as well as antibiotics and, notably, progesterone [8].

In non-IgE-mediated mast cell degranulation, receptors on mast cells are activated directly, or the allergen mimics IgE receptor binding. The clinical phenotype is indistinguishable from IgE-mediated disease, and current World Allergy Organization terminology accordingly classifies such events as non-IgE-mediated anaphylaxis rather than as anaphylactoid reactions, the older term having implied a distinct clinical entity that the phenotype does not support. Common triggers include contrast agents, opioids, progesterone, and progestins, and progesterone and progestin receptors have been identified directly on mast cell surfaces [6].

Typical presentation of PH

PH typically occurs in premenopausal women or during pregnancy [5]. Endogenous progesterone peaks during the luteal phase, when it is secreted by the corpus luteum to maintain the secretory endometrium; withdrawal of progesterone at luteolysis then precipitates endometrial shedding. Prior cases document heterogeneous clinical presentations, including papulopruritic rash and mucocutaneous angioedema consistent with PH, occurring cyclically approximately one week before menses [3,5]. Pregnancy-associated cases correlate with increased progesterone secretion from the corpus luteum supporting placental growth. Exogenous progestin use in oral or intrauterine contraceptives and in fertility treatment has likewise been associated with PH.

Only one male case of PH is documented in the peer-reviewed literature. In a 1996 correspondence, Fisher described a man with AIDS who developed papulopruritic urticaria and dermatitis after taking megestrol acetate for appetite stimulation and weight gain, confirmed by a positive progesterone skin-prick test [4]. Historically, diagnosis of PH required a clinical history of progesterone or progestin exposure, a cyclic rash aligned with ovulation, a positive progesterone skin-prick test, and physical findings such as atopy or respiratory symptoms [5]. Fisher challenged these criteria so that men could be diagnosed without the cyclical criterion [4]. However, details regarding treatment, management, and prognosis were not available for that patient.

Management of PH in the critical care setting

Our patient's combination of papulopruritic urticaria, mucocutaneous angioedema, and a previously positive progesterone skin-prick test satisfies the historical diagnostic criteria for PH as modified for male patients, notwithstanding the absence of any exogenous progestogen exposure [4,5]. The limitations of that diagnostic basis are addressed below.

In the emergency department, he received prompt intramuscular epinephrine, which remains the first-line treatment for severe allergic reactions and for anaphylaxis [7]. Coronary artery disease is not a contraindication to intramuscular epinephrine in anaphylaxis, and current guidance is explicit that epinephrine should not be withheld from patients with cardiovascular disease when anaphylaxis is present, since untreated anaphylaxis itself imposes substantial myocardial risk [7]. In this patient, the decision to withhold a third dose was individualized rather than categorical: he developed chest pain after the second dose, and neither hypotension, hypoxemia, bronchospasm, nor stridor was documented at that time. In the absence of cardiovascular or respiratory compromise, the anticipated benefit of further epinephrine did not outweigh the risk of provoking demand ischemia, and he was instead transferred for continuous cardiac and airway monitoring where epinephrine could have been resumed immediately had he deteriorated. Had hypotension, hypoxemia, or airway compromise developed, further epinephrine would have been indicated regardless of his coronary disease.

Upon ICU admission, the patient continued to experience intense pruritus and discomfort, managed immediately with ice pack application. Additional treatments included dexamethasone for inflammation, hydromorphone for pain management, and continuation of his home regimen of cromolyn sodium, which inhibits mast cell degranulation and histamine release. Resolution of symptoms following these interventions is consistent with, though not diagnostic of, a type I hypersensitivity reaction. Heffler et al. reported successful use of omalizumab, a monoclonal anti-IgE antibody, in a patient with recurrent progesterone-related anaphylaxis refractory to antihistamines and corticosteroids, with sustained remission on treatment [12]. Omalizumab is attractive in this setting because it lowers free IgE and downregulates FcεRI expression on mast cells and basophils, addressing the presumed effector pathway rather than the trigger, and because it does not depend on suppressing an ovulatory cycle - the mechanism underlying most established options, which are inapplicable to a male patient. It is not curative, requires indefinite administration, and has not been studied prospectively in PH; nonetheless, given that our patient can no longer tolerate chronic corticosteroids, it is the most rational of the currently available options.

The patient had been prescribed intramuscular testosterone cypionate every 10 days by his primary care provider and reported an incomplete preceding dose. Testosterone does not directly suppress endogenous progesterone through negative feedback in men. Testosterone replacement has been reported to reduce the proinflammatory cytokines tumor necrosis factor alpha and IL-1β and to increase anti-inflammatory IL-10 in hypogonadal men [13], and it is conceivable that such an effect contributes to symptom control, but this has never been studied in PH, and no therapeutic role is established. His most recent injection was incomplete because of a syringe plunger malfunction, and his presentation approximately six days later falls within the interval over which levels would be expected to decline given the roughly eight-day half-life of testosterone cypionate [14]. We emphasize that this is a temporal association in a single patient and not evidence of causation; the episode may equally have occurred independently of the missed dose, and no inference should be drawn that testosterone prevents PH.

Possible underlying clinical mechanisms in a male patient

Our case centers primarily on ICU management, with the diagnosis of PH based on physical findings, clinical history, and previous progesterone skin testing. If endogenous progesterone triggered our patient's reaction, several theories may be advanced, each of which is speculative and none of which was tested during this admission.

First, the patient had an intestinal carcinoid tumor discovered during a colonoscopy after his initial presentation with PH in 2006. His last colonoscopy was more than two years prior, exceeding the recommended surveillance interval [15]. Intestinal carcinoid tumors, typically associated with serotonin hypersecretion, have not been definitively linked with progesterone expression; however, progesterone receptor expression has been described in carcinoid tumors of the kidney [16]. Our literature search identified no previously reported association between carcinoid tumors and PH, and the link considered here is therefore hypothetical rather than established. Reevaluation for recurrent carcinoid disease, together with measurement of progesterone levels, may nonetheless be reasonable, particularly as his last colonoscopy preceded the recommended surveillance interval.

Normal endogenous progesterone in men is 0.1-1.0 ng/mL, comparable with levels in postmenopausal women and substantially lower than in women during the mid-to-late luteal phase (8.6-19.9 ng/mL) [17,18]. 17-hydroxyprogesterone, synthesized in the adrenal glands by 17α-hydroxylase, is normally present in lower quantities [19]. Elevated 17-hydroxyprogesterone, as seen in nonclassic or late-onset congenital adrenal hyperplasia, may provoke a type I hypersensitivity reaction and would justify adrenal evaluation [20].

Limitations

This report has important limitations that bear directly on the certainty of the diagnosis. It is a retrospective description, and serial vital signs and detailed cardiopulmonary examination findings were not available to us for reporting; the absence of documented hypotension, hypoxemia, and bronchospasm is therefore based on the clinical record as available rather than on a prospectively completed dataset. No serum tryptase was obtained during the acute episode, and no basal tryptase has been measured; acute and basal tryptase levels are central to distinguishing mast-cell-mediated reactions from mimics, and their absence is a major diagnostic limitation. No serum progesterone or 17-hydroxyprogesterone level was obtained, so the mechanistic hypotheses advanced above regarding endogenous progestogen excess and adrenal hyperplasia remain untested. No bone marrow evaluation, KIT D816V testing, or serum IgE quantification was performed, and chronic spontaneous urticaria and mast cell activation syndrome were therefore not formally excluded. The diagnosis rests on the clinical history, the physical findings described, and a progesterone skin-prick test performed at an outside institution years earlier, whose methodology could not be verified - a particular concern because progesterone skin testing is not standardized and false-positive results attributable to diluents such as benzyl alcohol and oil bases are well described [3]. We could not verify whether any agent beyond those listed was administered when he developed chest pain. These investigations should be regarded as the appropriate next steps in the outpatient evaluation of this patient and of any male patient in whom PH is suspected, rather than as criteria that were met in this case.

Finally, misdiagnosis or the need for further evaluation remains possible given the rarity of PH. The largest series of PH, comprising 24 patients from Brigham and Women's Hospital, found false-positive skin-prick tests attributable to diluents such as benzyl alcohol or oil bases, and only half of clinically consistent cases yielded positive tests [3]. Consequently, differential diagnoses such as hyper-IgE syndrome, chronic spontaneous urticaria, and mast cell activation syndrome must be considered, and in this patient, they were not formally excluded, as detailed above. ICU management would nonetheless have remained unchanged, since the treatment delivered was directed at symptom control rather than at a specific mechanism.

## Conclusions

This case highlights a rare presentation of PH in a male patient treated effectively in the emergency department and the intensive care unit. Limited information is available on diagnosing and treating this condition because of its extreme rarity, particularly in male patients. Prompt recognition and initiation of therapy, including epinephrine, corticosteroids, and supportive care, are critical to relieving acute symptoms and preventing progression to anaphylactic shock. Consistent outpatient follow-up testing to further clarify the underlying cause and individual pathophysiology remains appropriate in both men and women. While this case illustrates one approach to the management of a severe hypersensitivity reaction attributed to PH in a man, the diagnosis rests on incomplete objective data, and the mechanisms proposed here remain speculative. Each presentation requires individual clinical judgment, and confirmatory testing should be pursued wherever possible.
